# Application of veno-arterial-venous extracorporeal membrane oxygenation in differential hypoxia

**DOI:** 10.1186/2049-6958-9-55

**Published:** 2014-11-04

**Authors:** Joon Hyouk Choi, Su Wan Kim, Young Uck Kim, Song-Yi Kim, Ki-Seok Kim, Seung-Jae Joo, Jung Seok Lee

**Affiliations:** Deparment of Cardiology, Jeju National University Hospital, Jeju National University School of Medicine, Jeju, Korea; Department of Thoracic and Cardiovascular Surgery, Jeju National University Hospital, Jeju National University School of Medicine, Aran 13 gil 15, Jeju-si, Jeju Special Self-Governing Province 690-767 Korea; Department of Neurology, Jeju National University Hospital, Jeju National University School of Medicine, Jeju, Korea

**Keywords:** Brain, Extracorporeal membrane oxygenation, Lungs, Pulmonary function

## Abstract

Veno-arterial extracorporeal membrane oxygenation (ECMO) through the femoral vein and artery may cause differential hypoxia, i.e., lower PaO_2_ in the upper body than in the lower body, because of normal cardiac output with severe impairment of pulmonary function. Hereby, we report the diagnosis and the treatment of differential hypoxia caused by veno-arterial ECMO. A 39-year-old man received cardiopulmonary resuscitation from a cardiac arrest due to acute myocardial infarction. Even after more than 30 min of resuscitation, spontaneous circulation had not resumed. Next, we performed veno-arterial ECMO through the femoral artery and vein, and the patient recovered consciousness on the second day of ECMO. On day 5 of ECMO, he lost consciousness again and presented a generalized tonic-clonic seizure, and an electroencephalogram showed delta waves suggesting diffuse cerebral cortical dysfunction. While an echocardiogram revealed improvements in myocardial function, a follow up chest radiograph showed increasing massive parenchymal infiltrations, and gas analysis of blood from the right radial artery revealed severe hypoxemia. These findings indicated a definite diagnosis of differential hypoxia, and therefore, we inserted a 17-Fr cannula into the left subclavian vein as a return cannula. The patient’s consciousness and pulmonary infiltrations were improved 2 days after veno-arterial-venous ECMO, and the electroencephalogram showed normal findings. To our knowledge, this is the first report of successful clinical management of differential hypoxia. We suggest that veno-arterial-venous ECMO could be the treatment of choice for differential hypoxia resulting from veno-arterial ECMO.

## Background

Femoral veno-arterial extracorporeal membrane oxygenation (VA ECMO) may cause differential hypoxia (lower PaO_2_ in the upper body than in the lower body, i.e., two-circulation syndrome) because of normal cardiac output with severe impairment of pulmonary function [1]. The phenomenon of differential hypoxia has been introduced theoretically; however, to our knowledge, thus far no definitive clinical report has been published on the management of differential hypoxia.

Hereby, we report a case of differential hypoxia resulting from VA ECMO and its successful treatment using veno-arterial-venous (VAV) ECMO in a patient with respiratory failure resulting from cardiac arrest.

## Case presentation

**Figure 1:**
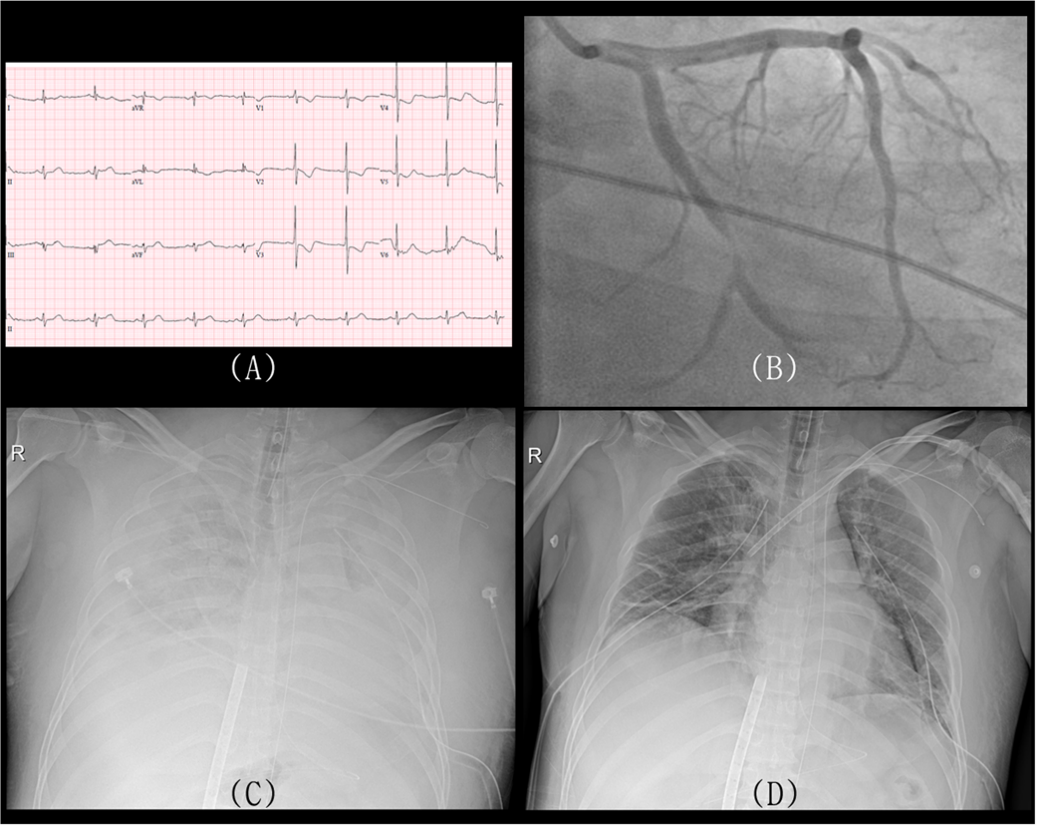
**Images showing clinical progression of differential hypoxia.** Electrocardiogram shows depressions of the ST segment in V1 to V6 leads **(A)**. Coronary angiography reveals a simple lesion in the left circumflex coronary artery **(B)**. Severe pulmonary edema and bilateral pleural effusions are observed on the chest radiograph **(C)**. Pulmonary edema was improved after veno-arterial venous extracorporeal membrane oxygenation **(D)**.

A 39-year-old man presented with chest pain that had lasted for 90 minutes. Electrocardiography revealed ST segment depression in leads V1–6 (Figure 1A). Five minutes after his arrival at the emergency department, he lost consciousness and displayed indications of a seizure. The electrocardiogram showed alternate ventricular tachycardia and ventricular fibrillation. Defibrillation and cardiopulmonary resuscitation were immediately performed. Although resuscitation was performed for more than 30 minutes, spontaneous circulation had not recovered. A VA ECMO system (EBS, Capiox Emergency Bypass System; Terumo Inc., Tokyo, Japan) was set up with a 21-Fr drainage cannula (DLP, Medtronic Inc., Minneapolis, MN) from the left femoral vein and a 17-Fr infusion cannula into the left femoral artery, and a coronary angiography was performed (Figure 1B). Unexpectedly, coronary angiography showed a simple lesion in the left circumflex coronary artery, which did not occupy a large territory. After percutaneous coronary intervention of the lesion, an echocardiography, performed in the intensive care unit, showed severe left ventricular systolic dysfunction (ejection fraction =15%) with global hypokinesia of the myocardium. Severe pulmonary infiltrations and bilateral pleural effusions were observed on a chest radiograph (Figure 1C).

**Figure 2:**
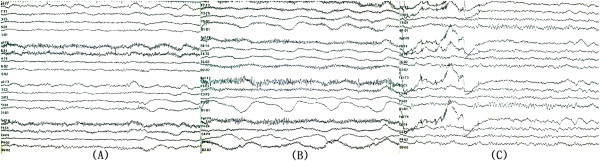
**Conversion of electroencephalogram.** At the time of admission electroencephalogram showed generalized reactive theta slowing **(A)**. On the fifth day of extracorporeal membrane oxygenation support, generalized delta activity with superimposed beta frequency was evident **(B)**. The electroencephalogram returned to normal configurations after veno-arterial venous extracorporeal membrane oxygenation **(C)**.

Pulmonary infiltrates were observed, which may be due to pulmonary edema resulting from cardiac arrest, considering that no history of suspicious pneumonia, idiopathic pulmonary fibrosis, or vasculitis was present. Furthermore, his chief complaint was chest pain, not dyspnea, and severe left ventricular systolic dysfunction was found by echocardiography. The patient showed stuporous mentality. An electroencephalogram (EEG) revealed bilateral theta waves of 5 to 6 Hz (Figure 2A). On the second day of ECMO, the patient regained consciousness, and inotropic agents were reduced. The arterial gas analysis of blood from the right radial artery revealed normal results (PaO_2_ 76 mmHg, O_2_ saturation 96.2%). Therefore, the ventilator settings were as follows: pressure control mode, inspired oxygen fraction 0.4, positive end-expiratory pressure 5 cm H_2_O, inspiratory pressure 16 cm H_2_O, and respiratory rate 14 breaths/minutes.

On the fifth day of ECMO, the patient lost consciousness again and displayed signs of generalized tonic-clonic seizure. Anticonvulsants were used immediately. The vital signs were stable, and a follow up echocardiogram revealed improvements in myocardial function and left ventricular ejection fraction (50%). A follow up chest radiograph revealed severe pulmonary edema, and arterial gas analysis of blood from the right radial artery revealed severe hypoxemia (PaO_2_ 39 mmHg, O_2_ saturation 69.4%). At that time, the patient was in comatose mentality. EEG showed relatively fast beta frequency activity superimposed on delta slow waves, which implied that cerebral cortical function had further deteriorated (Figure 2B). The ventilator settings were changed as follows: pressure control mode, inspired oxygen fraction 1.0, positive end-expiratory pressure 10 cm H_2_O, inspiratory pressure 24 cm H_2_O, and respiratory rate 24 breaths/minutes. However, right radial hypoxemia did not recover.

These findings indicated a definite diagnosis of differential hypoxia, and we decided to perform VAV ECMO. A 17-Fr cannula was inserted into the left subclavian vein. Venous blood from the femoral vein was drained and oxygenated by ECMO and then perfused into the femoral artery and the left subclavian vein. The oxygenated blood entered the right atrium and bypassed the non-functioning lungs. Finally, the oxygenated blood reached the brain through the left ventricle and ascending aorta, and then right radial hypoxemia was recovered (PaO_2_ 103 mmHg, O_2_ saturation 98.9%). The patient recovered consciousness and pulmonary infiltrations were improved 2 days after VAV ECMO (Figure 1D). The patient’s EEG at that time was normal (Figure 2C). ECMO and mechanical ventilator were removed 5 days after VAV ECMO. The patient was discharged 97 days after ECMO removal for rehabilitation, and followed up for 13 months without any cardiac or pulmonary problems.

## Discussion

ECMO is used in patients with severe cardiopulmonary failure who might otherwise die. Recent advances in catheters, bypass circuits, membrane oxygenators, and percutaneous cannulation techniques have made possible to start a closed-chest cardiopulmonary bypass outside of the operating room. Percutaneous cardiopulmonary support has produced varying degrees of success as a therapeutic option in various clinical settings, including cardiac arrest, respiratory failure, and special medical conditions [2–5].

While the benefits of ECMO are well known, awareness regarding the complications of ECMO is limited to infection, bleeding, and thrombosis [6, 7]. Bleeding and thrombosis that occur regularly during the course of ECMO can result in significant clinical complications. Surgical site bleeding, the most common source of bleeding, is reported in 6–32% of ECMO patients, with the highest incidence observed in patients who have had recent cardiac surgery [8]. Intracranial hemorrhage, the most potentially devastating bleeding complication, occurs in 3–6% of patients. It is known that hypoxemia, acidosis, cardiovascular instability prior to initiation of ECMO, and coagulopathy increase the risk of intracranial hemorrhage [9].

**Figure 3:**
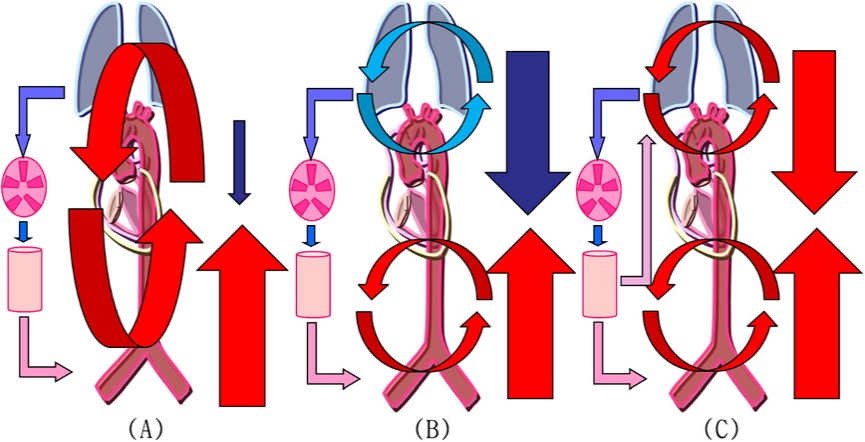
**Schematic diagrams of circulation during extracorporeal membrane oxygenation (ECMO).** The diagrams indicate circulation in the case of typical veno-arterial ECMO **(A)**, differential hypoxia **(B)**, and veno-arterial venous ECMO **(C)**. Red or pink arrows = oxygenated blood; blue arrows = deoxygenated blood; downward arrows = cardiac output; upward arrows = retrograde ECMO output.

A frequently overlooked complication of VA ECMO support is cerebral hypoxia. If the blood is infused into the femoral artery and flow is retrograde, fully saturated blood from the ECMO circuit comes into contact and mixes with blood ejected from the native ventricle in the mid aorta. The location of this mixing point depends on the amount of ECMO support provided and the degree of left ventricular ejection. In case of severe myocardial dysfunction, the mixing point is in the proximal ascending aorta or aortic root (Figure 3A). As myocardial function improves, the mixing point may migrate more distally into the aortic arch. During severe respiratory failure due to pulmonary edema, the typical VA ECMO flow rate (80% of full cardiac output) can result in desaturated blood from the left ventricle perfusing the aortic arch and coronary arteries and fully saturated infusion blood perfusing the lower body. The patient’s head appears blue, whereas the lower extremities appear pink (Figure 3B). This phenomenon is known as differential hypoxia or two-circulation syndrome [10–13].

Differential hypoxia is diagnosed on the basis of the finding of hypoxic arterial blood gas when saturations in the right radial artery are measured, which reflects the patient’s cardiac output. Because of the misconception that the lungs are unimportant while the patient is supported by VA ECMO, ventilator settings are frequently reduced to the minimum setting. Recently, Hoeper et al. [14] described a few images of an ECMO watershed. These images showed a watershed, where well-contrasted blood from the ECMO circuit met low-contrasted blood from the left ventricle resulting from acute pulmonary thromboembolism. Although the clinical course is similar, we think that differential hypoxia is different from ECMO watershed, because watershed is caused by a high preload (anatomical obstruction) and decreased left ventricular systolic function, whereas differential hypoxia is caused by a high afterload (physiological obstruction) and recovered left ventricular systolic function.

Variable methods to overcome differential hypoxia have only occasionally been described in the literature. These methods include the following: 1) ventilator adjustments, including increased oxygen supplementation and positive end-expiratory pressure [13], 2) increasing ECMO flow with full drainage of the superior vena cava [12], 3) inserting the arterial cannula into the carotid artery, instead of the femoral artery, or using a long cannula that could reach the aortic arch [11], and 4) considering central cannulation or perfusion via a subclavian vascular graft [10]. In our patient, we employed VAV ECMO, adding a 17-Fr return venous cannula into the left subclavian vein. The oxygenated blood from the circuit of the femoral artery perfused into the left subclavian vein and then into the right atrium and pulmonary arteries. The oxygenated blood bypassed the damaged lung and perfused the coronary arteries and aortic arch (Figure 3-C). Importantly, the saturated blood entered the cerebrum and assisted recovery from hypoxic brain damage. VAV ECMO itself was not a method for left ventricular decompression. However, it supplied oxygenated blood to the vital organs in the upper body, especially the brain and myocardium, and then it presented a possibility for weaning ECMO. Simultaneous VAV ECMO and decreasing ECMO flow resulted in decreased afterload of the left ventricle. It can lead to a decrease in left atrial pressure, resulting in improvement of pulmonary edema.

## Conclusions

Differential hypoxia has been theoretically described in the literature; however, no case involving its clinical management has been reported thus far. To our knowledge, this report is the first which describes the diagnosis and the treatment of differential hypoxia, in which hypoxic brain damage was identified through EEG monitoring and resolved with VAV ECMO. If differential hypoxia resulting from VA ECMO for myocardial infarction is left undiagnosed, it can lead to weaning failure from ECMO, which could be fatal. Therefore, during VA ECMO, saturation levels in the right radial artery must be assessed for differential hypoxia. Furthermore, we believe that VAV ECMO could be the treatment of choice for differential hypoxia resulting from VA ECMO.

## Consent

Written informed consent was obtained from the patient for publication of this case report and any accompanying images. A copy of the written consent is available for review by the Editor-in-Chief of this journal.
